# JOURNAL OF GERONTOLOGY: BIOLOGICAL SCIENCES

**DOI:** 10.1093/geroni/igae098.0906

**Published:** 2024-12-31

**Authors:** Gustavo Duque

**Affiliations:** Bone, Muscle & Geroscience Group, Research Institute of the McGill University Health Centre, Montreal, Quebec, Canada

## Abstract

The “Journal of Gerontology: Biological Sciences” is a peer-reviewed academic journal that focuses on research related to the biology of aging. It covers a broad range of topics within this field, including genetics, cellular aging, molecular mechanisms of aging, age-related diseases, and interventions to promote healthy aging. More recently, we have participated in the creation of a Translational Geroscience Section, which integrates the growing subject of geroscience into both the medical and biological sections of the journal with an emphasis on the translation of discoveries into clinical practice. Our journal serves as a valuable resource for researchers, clinicians, and policymakers interested in addressing the challenges and opportunities associated with an aging population. In this session, the editorial criteria for selecting the highest-quality manuscripts will be discussed. In addition, practical tips for preparing manuscripts reporting biomedical research will be provided.

